# Effects of wet cupping in a rat model of primary dysmenorrhea

**DOI:** 10.1016/j.jaim.2024.101047

**Published:** 2024-12-09

**Authors:** Sri Lestariningsih, Didik Gunawan Tamtomo, Sri Sulistyowati, Dono Indarto, Soetrisno Soetrisno, Hanik Badriyah Hidayati, Wahyudi Widada

**Affiliations:** a Doctoral Program of Medical Sciences, Faculty of Medicine, Universitas Sebelas Maret, Surakarta, 57126, Indonesia; b Midwifery Program, Tanjungkarang Ministry of Health Polytechnic, Metro City, Sumatera, Lampung, Indonesia; c Department of Obstetrics and Gynecology, General Hospital UNS/Faculty of Medicine, Universitas Sebelas Maret, Surakarta, Indonesia; d Department of Physiology, Faculty of Medicine, Universitas Sebelas Maret, Surakarta, Indonesia; e Biomedical Laboratory, Faculty of Medicine, Universitas Sebelas Maret, Surakarta, Indonesia; f Department of Neurology, Faculty of Medicine Airlangga University, Dr. Soetomo General Hospital, Mayjend. Prof. Dr. Moestopo Street, Number: 6-8, Surabaya, East Java, 60286, Indonesia; g Faculty Health of Science, Muhammadiyah University of Jember, Jember, East Java, Indonesia

**Keywords:** Pain suppression, Uterine morphology, Rat model, Primary dysmenorrhea

## Abstract

**Background:**

Primary dysmenorrhea (PD) is characterized by discomfort with no organic etiology (no pelvic disease), recurring pain, or lower abdominal cramps that start between the first 8–72 h of menstruation. Cupping therapy uses a tool to form a vacuum at certain points on the skin.

**Objectives:**

We investigated the mechanism of pain relief caused by cupping therapy in primary dysmenorrhea that is played by cupping therapy in PD. This study aimed to investigate the effects of the cupping method on pain symptoms, changes in PGF2α, PGE, and β-endorphin levels, and uterine morphology in PD.

**Methods:**

A total of 35 female rats were divided into five groups (n = 7 rats per group): control, PD, dysmenorrhea treated with dry cupping (DC), dysmenorrhea treated with wet cupping (WC), and dysmenorrhea treated with ibuprofen (IB) as a standard drug. Pain was assessed by measuring the degree of writhing pain. Serum PGF2α, PGE, and β-endorphin levels were evaluated using ELISA. Hematoxylin-eosin staining was used to examine uterine morphology, such as thickness, vacuolization, and inflammation.

**Results:**

WC had a pain normalization effect comparable to that of ibuprofen. Ibuprofen is superior to both types of cupping in reducing the PGF2α/PGE ratio and the PGF2α to β-endorphins ratio. WC and DC have capabilities comparable to those of ibuprofen in improving uterine vacuolization and inflammation.

**Conclusions:**

These results indicate that WC is more effective than DC in suppressing dysmenorrhea symptoms, modulating the hormone level ratio, and repairing uterine pathology. The potential benefits of cupping provide an opportunity for further studies in human subjects.

## 1 Introduction

Dysmenorrhea is a cyclical crampy sensation in the lower abdomen or pelvic pain that occurs before or during menstruation. The pain is spasmodic, originating in the uterus and spreading to the thighs or lower spine. Sufferers may experience feelings of discomfort, such as in the form of nausea, headaches, back pain, diarrhea, and fatigue during menstruation [1,2]. Primary dysmenorrhea (PD) is described as discomfort with no organic etiology (no pelvic disease), recurring pain, or lower abdominal cramps that start between the first 8–72 h of menstruation [1,3]. Secondary dysmenorrhea can happen at any age, from menarche to menopause, and is usually caused by gynecological pathologies as endometriosis, adenomyosis, uterine fibroids, and ovarian cysts [2,3].

According to the WHO, the incidence varies between 45% and 79% across various age groups and countries [4]. The prevalence of primary dysmenorrhea among university students is 95% in Indonesia, 89.10% in Iran, 85.4% in Ethiopia, 80.6% in Lebanon, 80.01%-85, 7% in Saudi Arabia, 64.0% in Mexico, and 41.7–51.1% in China [3,[5], [6], [7], [8], [9]]. Long and extended menstrual flow, young age, young age of menarche, nulliparity, smoking, and family history are risk factors for PD [10,11]. Primary dysmenorrhea is associated with a lower quality of life, depression, and anxiety [2].

Primary dysmenorrheic pain appears 6–12 months after menarche and persists until menopause. Moderate (37–47%) to severe (17–18%) pain is the main reason for a visit to a gynecologist [3,12]. While the exact cause of primary dysmenorrhea is still under investigation, a leading theory points to increased prostaglandin production and release. These prostaglandins cause the myometrium to contract excessively, leading to hypoxia and ischemia within the muscle, ultimately resulting in discomfort [10,13]. In addition, increased prostaglandin (PG), vasopressin, and oxytocin levels contribute to enhanced activation of pain in type C fibers [3].

A previous report indicated that the prostaglandins E2 and F2 play a role in the pathophysiology of PD. PGF2α induces uterine vasoconstriction, myometrial contractions, and uterine ischemia. An increase in PGF2α/PGE ratio is a sign of PD. High PGF2α levels bind to the PGF2 receptor (PTGFR), activating Cb phospholipases and triggering diacylglycerol (DAG) release DAG activation triggers protein kinase C (PKC) signaling, resulting in increase connexin 43 (CX43) and excessive uterine contractions [14]. The pain profile in dysmenorrhea is based on functional and anatomical alterations in pain-related regions of the brain. Pain therapy for dysmenorrhea prevents the long term onset of chronic pelvic pain [2].

PD can be treated either pharmacologically or non-pharmacologically. Pharmacological treatment are divided into non-hormonal and hormonal treatments. Non-hormonal treatments include acetaminophen, pamabrom, and NSAIDs [15]. NSAIDs are the first-line of therapy for PD [16]. However, gastrointestinal irritation, central nervous system disorders, renohepatohematotoxicity, bronchospasm, and resistance persists [17]. The rate of resistance to NSAIDs during PD treatment was 16%. Hormonal therapy comprises of a combination of oral contraceptives and progestins. If used repeatedly, progesterone can cause weight gain, menstrual problems, melasma, and amenorrhea [18,19]. Thus, the facts prove that the current PD treatment does not meet the needs of patients so that new therapeutic approaches are needed. One study reported that patients with PD self-medicate, seek medical treatment, or undertake complementary medicine [20].

Cupping therapy has been traditionally used since the year 2000. There are several cupping therapies, including needle cupping, dry cupping, bleeding cupping (wet cupping), and herbal cupping. Cupping therapy uses a tool to create a vacuum at certain points on the skin. This technique's ability to reduce symptoms has made it popular in many parts of the world [21]. Several studies have explained the positive effects of cupping by several designs [22]. Although the outcomes vary [23,24], cupping is available to treat a variety of pains, including neck and upper back pain [25], lower back pain [24,26], carpal tunnel syndrome [27], and osteoarthritis. Cupping is a common treatment used to treat metabolic pain [28]. However, the mechanisms of pain relief in each of these disorders has not been fully elucidated in primary dysmenorrhea. This study aimed to investigate the mechanism of pain relief caused by in PD.

## 2 Material and methods

### 2.1 Animals

Fifty female *Sprague Dawley* rats were acclimatized to laboratory conditions for 7 d. After acclimatization, estrous cycle screening was carried out for 7 days. Rats with regular estrous cycles were randomly selected for this study. The number of rats selected was 35, which were divided into five groups, namely, control, PD, PD that received dry cupping (DC), PD that received wet cupping (WC), and primary dysmenorrhea receiving ibuprofen as standard medication (IB).

Healthy, active, females, aged between 8 and 10 weeks, weighing 200–250 g were selected. Acclimatization and maintenance were performed in laboratory conditions (temperature of 23–25 °C and humidity of 50–60 %). A light/dark cycle was created for 12 h with a lighting intensity of 300 l. The rats were fed standard food (Biorat) and provided with drinking water via reverse osmosis ad libitum.

### 2.2 Creating a primary dysmenorrhea model

An animal model was established using previousl described methods [29]. Acclimatized rats in the estrous cycle will be injected (subcutaneously) with 5 mg of estradiol benzoate on the first and tenth day. Subsequently, 3 mg estradiol benzoate was applied from the second to nineth day. An intraperitoneal injection of 3 U oxytocin was performed on the last day.

### 2.3 DC method

DC therapy was given on the 2nd, 5th, and 10th days. The 10th day of therapy was administered shortly before oxytocin administration. The first cupping was done with a cup with a diameter of 2 cm and a negative pressure of −0.04 MPa in the right and left back areas (bilateral side of L4/L5 vertebrae) for 5 min. In puncturing, 10 punctures are carried out using a needle (depth ≤0.1 mm). The second cupping method was performed for 5 min with negative pressure of 200 mmHg.

### 2.4 WC method

WC therapy was given on the 2nd, 5th, and 10th days. The 10th day of therapy was administered shortly before oxytocin administration. The first cupping was done with a cup with a diameter of 2 cm and a negative pressure of −0.04 MPa in the right and left back areas (bilateral side of L4/L5 vertebrae) for 5 min. Ten punctures are performed out using a 21 G needle (depth ≤0.1 mm). The second cupping method was done by cupping for 5 min with a negative pressure −0.04 MPa.

### 2.5 Measurement of writhing frequency

Pain symptom were detected by observing writhing for 20 min. Twisting reactions in rat models (abdominal contractions, concave, trunk and hindlimb extensions, rotation of one limb, and uterine contractions) indicate the availability of PD model [30].

### 2.6 Analysis of PGF2α, PGE2, and β-endorphine levels

An enzyme-linked immunosorbent assay (ELISA) technique was performed to measure PGF2α, PGE2, and *β*-endorphine levels. The rat PGF2 ELISA Kit (FINETEST cat: ER1257-96 wells; (Wuhan Fine Biotech Co., Ltd. China), Rat Prostaglandin E2 (PG-E2) ELISA Kit (Bioenzy cat: BZ08184050-EB), and Rat Beta-Endorphin ELISA Kit (Bioenzy cat: BZ-08187000-CPEB) were used. Analysis was performed per the manufacturer's protocol.

### 2.7 Histopathological analysis

The uterus was removed and submerged in 10% formaldehyde solution after the last induction. The tissues were dehydrated and eluted using an ethyl alcohol gradient ranging from 80 to 100%, followed by paraffin embedding. The wax block was thinned to 3–5 m and stained with hematoxylin and eosin (Sigma) for histological evaluation under an optical microscope (CX23, Olympus, Japan).

### 2.8 Statistical analysis

A non-parametric test was used to examine the tabulated data. Statistical significance was set at *p* < 0.05 was considered significant. Statistical analysis was performed using the SPSS software version 16.

## 3 Results

**Fig. 1:**
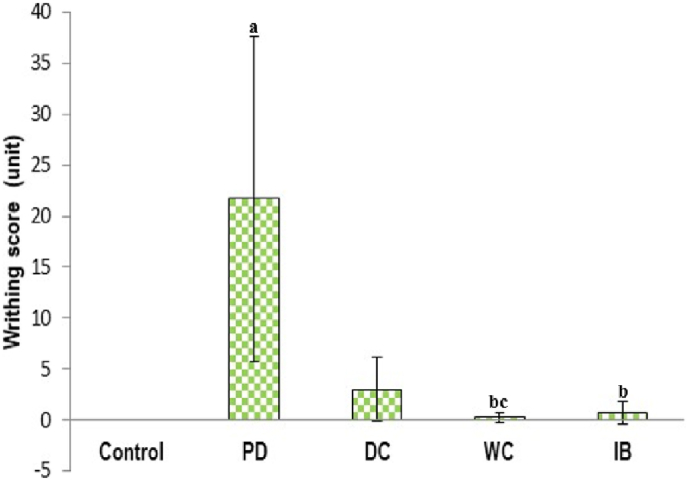
Frequency of writhing score. PD: primary dysmenorrhea; DC: the primary dysmenorrhea received dry cupping; WC: the primary dysmenorrhea group received wet cupping; IB: The primary dysmenorrhea group received ibuprofen as standard medication; ^a^: *p* < 0.05 in comparison to the control group; ^b^: *p* < 0.05 in comparison with the PD group; ^c^: *p* < 0.05 in comparison with the DC group.

Fig. 1 shows the writhing frequency of each group. Compared with the control group, there was a significant increase in the PD group (*p* < 0.05). The writhing frequencies in the DC and WC groups were significantly lower than those in the PD group (*p* < 0.05). We also found a significant decrease in the IB group compared to the PD group (*p* < 0.05). The frequency in the WC and IB groups did not change significantly from those in the control group (*p* > 0.05).

**Fig. 2:**
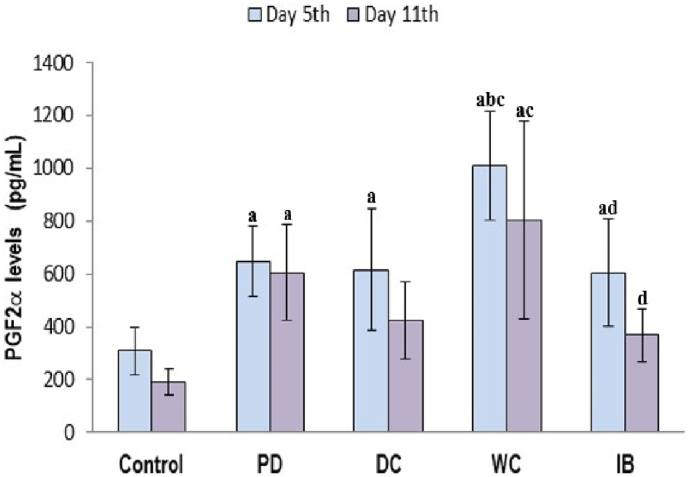
Serum PGF2α levels. PD: primary dysmenorrhea group; DC: the primary dysmenorrhea group received dry cupping; WC: the primary dysmenorrhea group received wet cupping; IB: The primary dysmenorrhea group received ibuprofen as standard medication; ^a^: *p* < 0.05 in comparison with the control group; ^b^: *p* < 0.05 in comparison with the PD group; ^c^: *p* < 0.05 in comparison with the DC group; ^d^: *p* < 0.05 in comparison with the WC group; pg/m: picogram/mililiter.

PGF2α levels in the various groups are shown in Fig. 2. For the 5th day, the PGF2α levels in the PD group were significantly higher than those in the control group (*p* < 0.05). This significant increase was also observed in the DC, WC, and IB groups (*p* < 0.05). In the WC group, there was a significant elevation in PGF2α levels than those in the PD group (*p* > 0.05). On the 11th day of observations a significant increase in PGF2α levels was observed in the PD group compared with the control group (*p* < 0.05). An insignificant decrease was observed in the PGF2α levels in the DC, WC and IB groups compared to the PD group (*p* > 0.05).

**Fig. 3:**
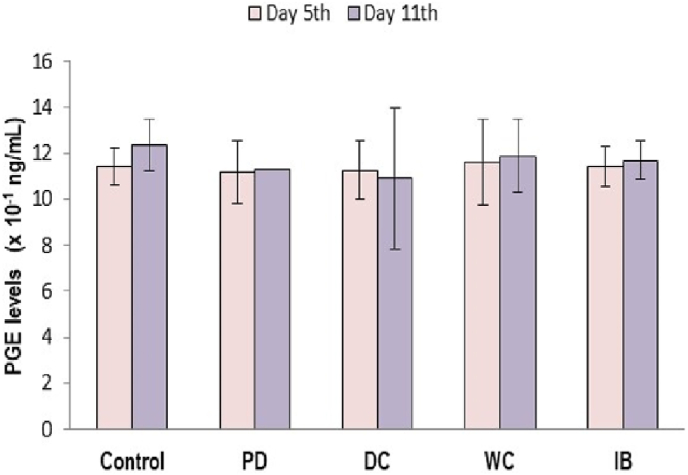
Serum PGE levels. PD: primary dysmenorrhea group; DC: the primary dysmenorrhea group received dry cupping; WC: the primary dysmenorrhea group received wet cupping; IB: The primary dysmenorrhea group received ibuprofen as standard medication; mg/mL: microgram/mililiter.

Fig. 3 shows serum PGE2 levels in the different study groups. On the 5th and 11th days of observation, there were no significant differences between the groups (*p* > 0.05).

**Fig. 4:**
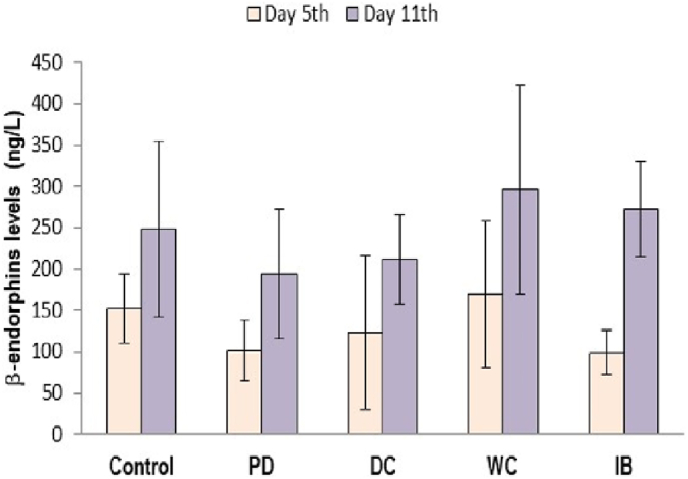
Serum β-endorphins levels in all groups. PD: primary dysmenorrhea group; DC: the primary dysmenorrhea group received dry cupping; WC: the primary dysmenorrhea group received wet cupping; IB: The primary dysmenorrhea group received ibuprofen as standard medication; ng/mL: nanogram/mililiter.

Fig. 4 shows the β-endorphins levels from various study groups. For day 5, β-endorphin levels were significantly lower in the PD group than those in the controls (*p* < 0.05). A significant increase was detected in the WC group compared to that in the PD group, reaching values comparable to those in control group (*p* < 0.05). Meanwhile, there were insignificant differences in the β-endorphins levels between groups on the 11th day of observation.

**Fig. 5:**
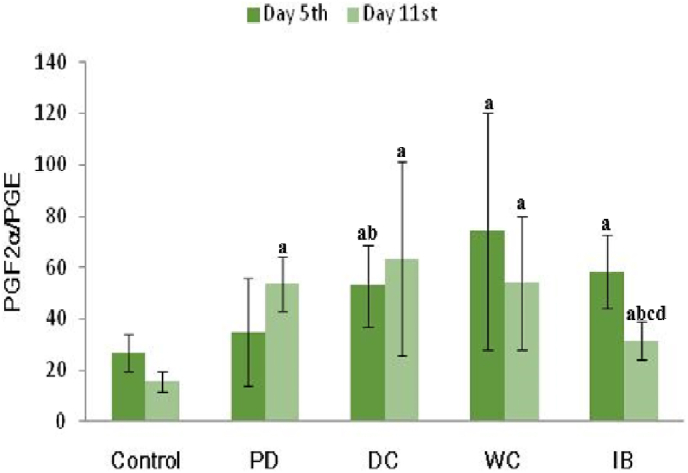
PGF2/PGE ratio. PD: primary dysmenorrhea group; DC: the primary dysmenorrhea group received dry cupping; WC: the primary dysmenorrhea group received wet cupping; IB: The primary dysmenorrhea group received ibuprofen as standard medication; ^a^: *p* < 0.05 in comparison with the control group; ^b^: *p* < 0.05 in comparison with the PD group; ^c^: *p* < 0.05 in comparison with the DC group; ^d^: *p* < 0.05 in comparison with the WC group.

Fig. 5 shows the PGF2α and PGE ratio. On the 5th day, there was no significantly difference in the ratio between the PD and the control groups (*p* < 0.05). On the 11th day, a significant increase in the PGF2α/PGE ratio between the PD and control groups (*p* < 0.05). AIl therapy significantly reduced the PGF2α/PGE ratio compared to the PD group (*p* < 0.05). This ratio was significantly lower in the IB group than in the DC and WC groups (*p* < 0.05).

**Fig. 6:**
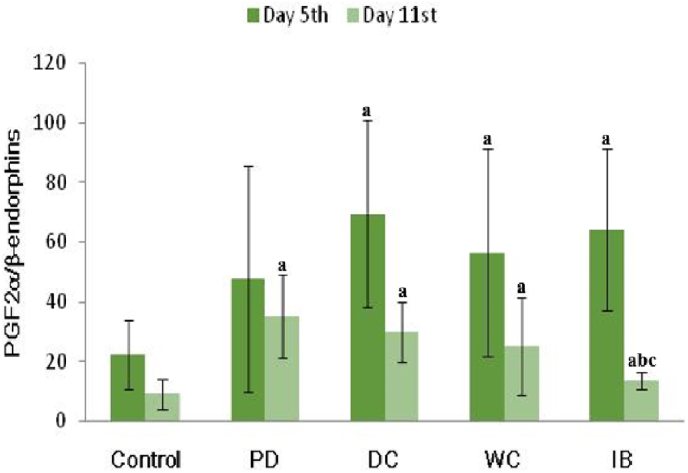
PGF2/β-endorphins ratio. PD: primary dysmenorrhea group; DC: the primary dysmenorrhea group received dry cupping; WC: the primary dysmenorrhea group received wet cupping; IB: The primary dysmenorrhea group received ibuprofen as standard medication; ^a^: *p* < 0.05 in comparison with the control group; ^b^: *p* < 0.05 in comparison with the PD group; ^c^: *p* < 0.05 in comparison with the DC group.

Fig. 6 shows the ratio of PGF2α to β-endorphins. No significant difference in the ratio of PGF2α/β-endorphins was observed on the 5th day, between the PD group and control groups (*p* < 0.05). On the 11th day, we found a significant increase in the ratio of PGF2α/β-endorphins in the PD group compared with that in the controls (*p* < 0.05). AIl therapy significantly reduced the ratio of PGF2α/β-endorphins significantly compared to the PD group (*p* < 0.05).

**Fig. 7:**
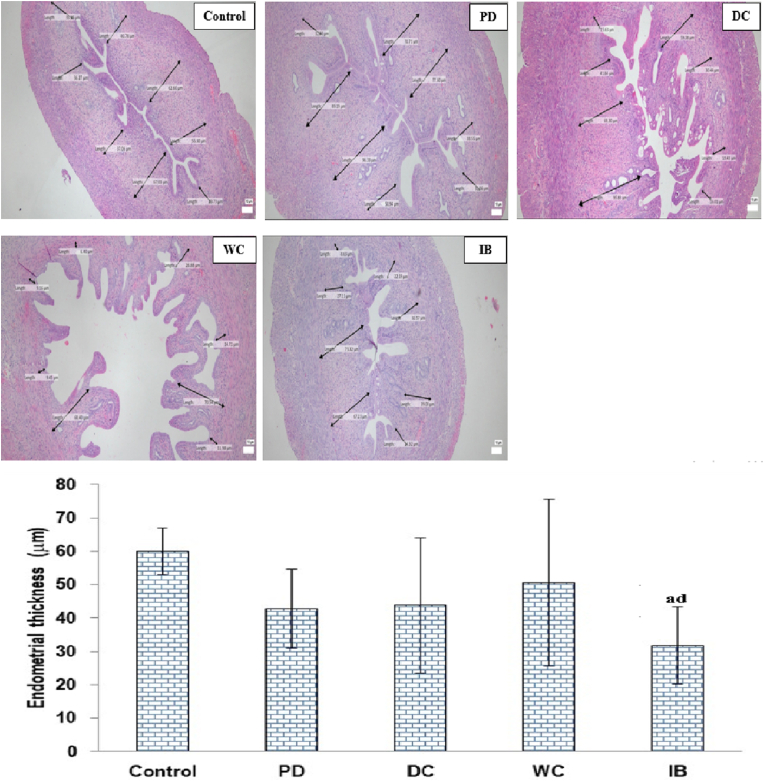
Endometrium thickness. Hematoxylin-eosin staining, magnification 400× (top figure); PD: primary dysmenorrhea group; DC: the primary dysmenorrhea group received dry cupping; WC: the primary dysmenorrhea group received wet cupping; IB: The primary dysmenorrhea group received ibuprofen as standard medication; ^a^: *p* < 0.05 in comparison with the control group; ^d^: *p* < 0.05 in comparison with the WC group (bottom figure).

Fig. 7 shows the endometrial thickness in each group. Endometrial thickness did not differ significantly between the PD group and control groups (*p* > 0.05). A non-significant difference was also found between the DC, WC, and IB groups and the control and PD groups (*p* > 0.05).

**Fig. 8:**
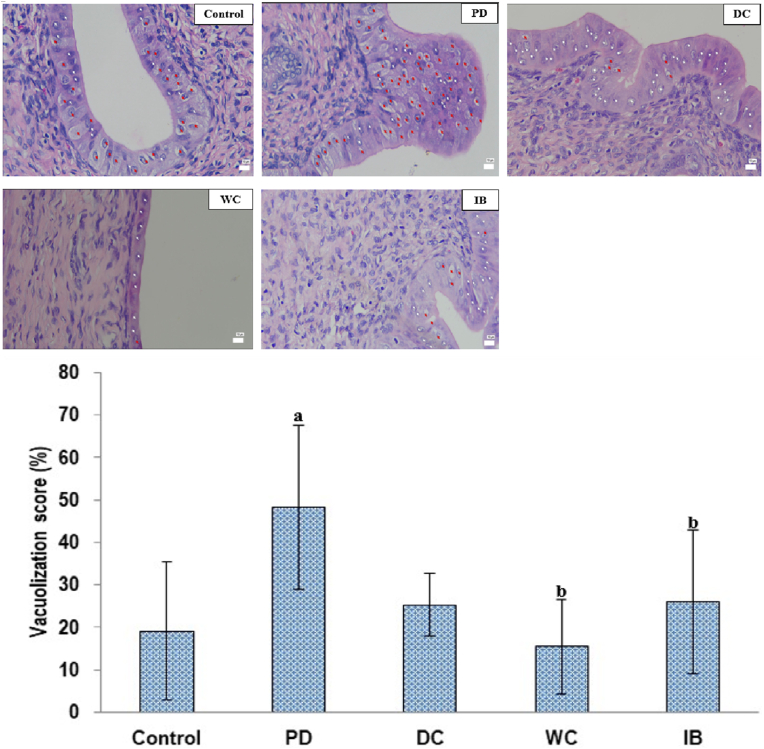
Cell vacuolization frequency. Red arrows: vacuolized cells; White arrows: cells that do not undergo vacuolization (Hematoxylin-eosin staining, magnification 400×) (top figure); PD: primary dysmenorrhea; DC: the primary dysmenorrhea received dry cupping; WC: the primary dysmenorrhea received wet cupping; IB: The primary dysmenorrhea group received ibuprofen as standard medication; ^a^: *p* < 0.05 in comparison with the control group; ^b^: *p* < 0.05 in comparison with the PD group; %: percentage (bottom figure).

Fig. 8 shows the degree of vacuolization in the distinct groups. The PD group demonstrated considerably more vacuolization than the control group (*p* < 0.05). This increase was minimized in the DC, WC, and IB groups when compared to the PD group (p 0.05), with the three groups reaching values comparable to those of the controls (*p* > 0.05). The degree of vacuolization did not differ significantly among the DC, WC, and IB groups (*p* > 0.05).

**Fig. 9:**
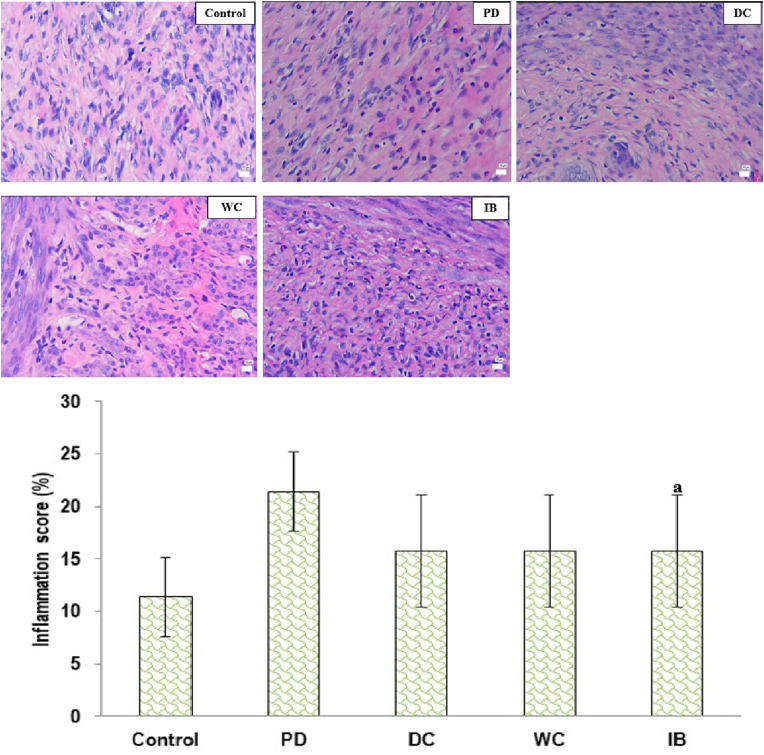
Degree of inflammation in the endometrium. Hematoxylin-eosin staining, magnification 400× (top figure); PD: primary dysmenorrhea; DC: the primary dysmenorrhea received dry cupping; WC: the primary dysmenorrhea group received wet cupping; IB: The primary dysmenorrhea group received ibuprofen as standard medication; ^a^: *p* < 0.05 in comparison with the control group; %; percentage (bottom figure).

Fig. 9 depicts uterine inflammation in each group. The PD group demonstrated significantly higher inflammation levels than the control group (*p* < 0.05). The degree of inflammation in the DC, WC, and IB groups was not significantly different from that in the PD group (*p* > 0.05).

## 4 Discussion

Our study showed that WC has a pain symptom normalization capacity comparable to that of ibuprofen. Ibuprofen is superior to both types of cupping in reducing the PGF2α/PGE ratio and the PGF2α to β-endorphins ratio. Wet and dry cupping have comparable capabilities to those of ibuprofen in improving uterine vacuolization and inflammation,

Elevated uterine prostaglandins levels cause dysmenorrhea. Prostaglandin production is inversely proportional to progesterone production. The model used in this study follows that of a previous study [29]. The two main regimens used for the induction of PD are estradiol benzoate and oxytocin. Estradiol benzoate, a natural estradiol derivative, enhances uterine smooth muscle cell contraction and increases oxytocin sensitivity. Oxytocin is a small peptide hormone that binds directly to oxytocin receptors in the cytoplasm of uterine smooth muscle cells. This binding generates prostaglandins, which increase excessive smooth muscle spasms, a feature of PD [14].

In this study, serum PGF2α levels increased in the dysmenorrhea group on the fifth and eleventh days, but there was no significant difference value in serum PGE levels between the groups. According to the theory, an increase in PGF2 indicates a response to estradiol benzoate, namely the contraction of uterine smooth muscle cells [14], although this was not proven in this study. PGF2α induces uterine vasoconstriction, myometrial contraction, and uterine ischemia. An increase rise in PGF2α/PGE ratio is a sign of primary dysmenorrhea. This increase in serum PGF2α levels is consistent with the results of previous study [23,31]. The lack of significance of serum PGE levels contradicts recent findings [32]. This increase in serum levels is linked to higher PGF2α levels in the uterus [33], as evidenced by reports in animal models and humans [[34], [35], [36]]. We found that on the eleventh day, there was a significant increase in the two ratios in the PD group compared to controls. This indicates that positive feedback was initiated by an increase in PGF2α, followed by an increase in PGE and β-endorphins. Our resullts are consistent with those of previous studies [14].

Increased PGF2α levels of in the uterus trigger uterine microvascular contractions, myometrial contractions, decreased uterine blood flow, hypoxia, ischemia and changes in uterine morphology [14]. In the study, changes in the uterine morphology were associated with increased vacuolization. Additionally, a decrease in endometrial thickness and an increase in inflammation also began to appear, although there were not statistically significant. Cell vacuolization is a morphological phenomenon that occurs in mammalian cells in the form of the appearance of vacuoles or vacuole-like structures [37]. Our study confirmed the findings of other studies demonstrating the emergence of vacuole-like degeneration in PD mouse models. The decreasing endometrial thickness in our model indicates a change in the endometrial lining to become disorganized, similar to previous findings of myometrial loss [38]. We found no significant difference in the endometrial thickness between the PD group and control groups. This differs from previous findings of thinning of the uterine wall and an increase in uterine diameter [30,39]. Human studies have found increased uterine thickness in three diameters and decreased endometrial thickness compared to controls [40]. Inflammation in PD mouse models is characterized by neutrophil infiltration, as observed in this study, although this is not statistically significant.

Uterine contractions and vasoconstriction associated with acute inflammation [38]. In contrast, menstruation is considered an inflammatory event, because leukocytic invasion and production of inflammatory mediators occurs during menstruation [41]. Uterine inflammation has also been observed in the PD model. This supports previous findings demonstrating an increase in inflammasomes in PD models [35], as well as the infiltration of inflammatory cells, especially neutrophils, in the uterus [38]. The inflammation generated by the dysmenorrhea model results from the metabolomic system of the body. Inflammatory molecules are elevated in patients with dysmenorrhea. Biliverdin, PGE, lactic acid, and docohexanoic acid (DHA) metabolites, on the other hand, are endogenous anti-inflammatory agents [42]. The PGE levels were insignificant in this investigation, indicating that PGE does not have an anti-inflammatory role.

β-endorphin is a hormone secreted by the pituitary glands. Plasma β-endorphin levels will increase when pain occurs in certain amounts and types. Next, β-endorphin will bind to the mu-opioid receptor. As an action outside the central nervous system, an increase in β-endorphin in peripheral tissues can affect the features of peripheral and local analgesia. In this study, β-endorphin levels in the DM group declined considerably on the 5th day compared to controls. This reduction is consistent with prior research [41]. However, on the eleventh day of observation, there were no significant changes in β-endorphin levels among the four groups, suggesting that the reduction in β-endorphin levels on the 5th day is evidence of modulation to peripheral tissues in the etiology of dysmenorrhea. Meanwhile, on the 11th day, β-endorphin's analgesic capacity vanished.

In this study, we applied two cupping techniques, namely, DC and WC. These two methods have been proven to significantly reduce writhing, which is clinical signs of dysmenorrhea. In reducing writhing, WC is more effective than DC alone and can even provide comparable effectiveness to NSAIDs. The mechanism for reducing pain is through increasing β-endorphins. Our findings are concistent with those of previous studies [43]. For morphological changes, both cupping methods and standard drugs were able to normalize uterine vacuolization, but were not able to improve inflammation. The application of WC triggers the stimulation of neurohormones and the immune system as well as the transfer of compounds in the microcirculation and interstitial fluid compartments [44].

Howewver, the limitation of this study was that there is no investigation at the genomic level to study the hormonal profile caused by dysmenorrhea or the opportunity for cupping to modify at the gene level. This will be of interest to future studies in animal models and humans.

## 5 Conclusion

WC was more effective than DC in suppressing dysmenorrhea symptoms. The efficacy of both types of cupping was comparable to that of standard drugs for improving uterine pathology. Thus, this study provides a solid foundation for studying of the benefits of cupping for symptom inhibition and organ repair in patients with dysmenorrhea.

## Author credit statement

Conception and design: SL, DGT, SS, DI, SS, WW

Collection and assembly of data: SL, DI, WW

Analysis and interpretation of data: SL, DI, SS, WW

Drafting the article: SL, SS, DI

Critical revisions of article for important intellectual content: DGT, SS, DI

Statistical expertise: SL

Final approval and guarantor of the article: DI

All authors have critically reviewed and approved the final draft and are responsible for the content and similarity index of the manuscript.

## Sources of funding

The authors did not received any specific grant from public, commercial, or non-profit sector.

## Declaration of generative AI in the writing process

The authors did not use any specific generative AI and AI-assisted technologies.

## Ethical approval and consent to participate

This study was evaluated and approved by the Medical and Health Research Ethics Committee, Faculty of Medicine, Public Health, and Nursing, Gadjah Mada University - Dr Sardjito Hospital, Yogyakarta, Indonesia (No. KE/FK/1453/EC/2022).

## Availability of data and material

The data and material will be provided on request.

## Conflict of interest

The authors declare the following financial interests/personal relationships which may be considered as potential competing interests:

Sri Lestariningsih reports administrative support, equipment, drugs, or supplies, and writing assistance were provided by 10.13039/501100007690Sebelas Maret University Faculty of Medicine. Sri Lestariningsih reports a relationship with 10.13039/501100007690Sebelas Maret University Faculty of Medicine that includes: non-financial support. Sri Lestariningsih has patent pending to None. Other authors declare that they have no known competing financial interests or personal relationships that could have appeared to influence the work reported in this paper.
